# Selective Laser Sintering of Nano Al_2_O_3_ Infused Polyamide

**DOI:** 10.3390/ma10080864

**Published:** 2017-07-27

**Authors:** Anthony Warnakula, Sarat Singamneni

**Affiliations:** Mechanical Engineering Department, Auckland University of Technology, Auckland 1010, New Zealand; awarnaku@aut.ac.nz

**Keywords:** selective, laser, sintering, polyamide, nano-ceramics, coalescence

## Abstract

Nano Al_2_O_3_ polyamide composites are evaluated for processing by selective laser sintering. A thermal characterization of the polymer composite powders allowed us to establish the possible initial settings. Initial experiments are conducted to identify the most suitable combinations of process parameters. Based on the results of the initial trials, more promising ranges of different process parameters could be identified. The post sintering characterization showed evidence of sufficient inter-particle sintering and intra-layer coalescence. While the inter-particle coalescence gradually improved, the porosity levels slightly decreased with increasing laser power. The nano-filler particles tend to agglomerate around the beads along the solid tracks, possibly due to Van der Walls forces. The tensile stress results showed an almost linear increase with increasing nano-filler content.

## 1. Introduction

Polymer nano-composites are made up of a matrix polymer, which is reinforced by a small quantity of nanoparticles with a high aspect ratio [1], and the particles or fibres used have a minimum of one dimension in the range of 1 to 100 nm [2]. Filler materials have been used with different polymers to improve properties such as mechanical strength, heat resistance, impact resistance, and electrical conductivity. Conventional fillers, which are in the macro or micro scale, lack strong interactions with the matrix polymer, and larger fillers are prone to imperfections [3,4]. Conventional fibre-reinforced composites with strong filler-matrix interactions show improvements in stiffness and strength while sacrificing toughness. The volume fractions of filler materials also have direct relationships to possible improvements in properties [2]. The presence of nano fillers in the matrix is more discrete in comparison, and adds to the properties of the base polymer without compromising on qualities such as flexibility. Layered silicates and carbon nanotubes are by far the most investigated nano materials [5].

The traditional methods used to process nano-composites can be divided into different sub categories. In the mechanical processing of nano materials, severe plastic deformation techniques such as forging, extrusion, drawing, and rolling are employed. The material is subjected to deformation under large strains below the recrystallization temperature [6,7,8]. These conventional techniques have limitations when processing nanoparticles due to load limitations, and it is difficult to achieve uniform microstructures. The formation of impurities and porosity is another problem associated with plastic deformation. Other techniques, such as ball milling, are used for processing, but lack the ability to produce bulk samples [6,7,9]. Shock wave consolidation is also used to process nanomaterials, which can densify powder materials without changing the microstructure or composition, unlike the thermo-mechanical processing methods.

In this context, the additive manufacturing routes, and in particular selective laser sintering (SLS), appear to offer promising solutions. In SLS, the polymer powder is deposited and consolidated layer by layer to produce complex and intricate three-dimensional parts [10]. The energy required for consolidation is supplied by a focused laser beam directed by a series of mirrors. The laser scanning path is determined by a computer aided design (CAD) model of the part. The sintering process can be characterised by different binding mechanisms used to process various materials. These mechanisms can be classed as solid state sintering, chemically induced binding, and liquid phase sintering. Solid state sintering occurs when the process temperature is from half of the melting temperature to the melting temperature. Diffusion is identified as the most prominent reaction under these conditions. This results in neck formation between adjacent powder particles due to the lowering of free energy. Most materials can be processed by solid state sintering, provided that the temperature is high enough to generate the kinetic energy required for the migration of vacancies. The speed of the process is dependent on the preheating conditions [11].

SLS has the ability to produce multilayer nano-composites, as it is a layer by layer production method employing localized heating. Studies by Zhang et al. [12] showed that the toughness of parts increases with smaller particle sizes. The study compares the differences between clay nanoparticle-modified nylon and standard nylon-6. Organic composites containing Carbon or hydrocarbon bonds as the backbone of the composition attained much research attention initially. Paggi et al. [13] researched a nano-composite comprised of polyamide 12 (PA12) and multi-walled carbon nanotubes (MWCNTs) developed using commercial material components. The polymeric component of the composite is Polyamide Duraform in powder form, and the multi-walled Carbon nanotubes were manufactured by chemical vapour deposition. The materials were mixed using a magnetic stirring technique to achieve homogenization. The specimens produced using SLS are used to identify the process parameters necessary to achieve high mechanical properties suitable for aerospace applications. It has been found that the material’s density and flexural modulus can be improved by adjusting the laser energy density. The study shows that the amount of carbon nanotube which could be mixed in the compound is limited to achieve proper sintering characteristics. The properties of PA12/MWCNTs were further investigated by Gean et al. [14]. The study revealed an increase in energy absorption in the presence of carbon nanotubes (CNTs) and an increase in tensile strength by 10%, while giving a reduction in elongation by 11–9%. A 12% increase in storage modulus was achieved. Polyamide 12 has also been combined with 3 wt % carbon nanofibers for processing using selective laser sintering [15]. The microstructural and mechanical characteristics have been evaluated using scanning electron microscopy (SEM) and dynamic mechanical testing. The micrographs have shown that the nanofibers are well-mixed in the polymer matrix of the sintered parts. The results show a 22% increase in the storage modulus from the original material. The composite has the potential for future processing if the powder’s characteristics are sufficiently altered.

Inorganic composites are also used in a range of applications, from producing functionally graded parts to producing biodegradable scaffold structures used in tissue engineering. In a study conducted by Chung et al. [16], Pure Nylon was blended with silica nanoparticles in the range of 2–6% by volume using a rotary tumbler to determine the effects of different loading and process parameters on the quality of the parts produced. The preferred powders were in the range of 10 to 150 micrometers in diameter, with semi-crystallinity and a low melting point. Previous research conducted on a micro Al_2_O_3_ particle-filled polyamide showed potential, but the micro ceramics adversely affected the toughness and flexibility of the material. Evidently, nano-fillers are more discrete due to the significant particle size differences, and are likely to contribute to material enhancements without adverse effects. Selective laser sintering is possibly the better way of consolidating the combined powders. The current research is an attempt in this direction, considering Al_2_O_3_ as the filler material, considering inherent characteristics such as high hardness, thermal stability, insulation, and flame retardancy, while polyamide is the base polymer. Al_2_O_3_ is proven for its high thermal stability, which allows the particles to absorb laser energy and release it at a slower rate compared to the polymer particles. The slow release in energy improves the solid state sintering of polymer, which is dependent on time and temperature. Nano ceramic particles were chosen instead of micro particles, as their presence in the polymer matrix is negligible.

## 2. Materials and Methods

Powder preparation is an important aspect in the selective laser sintering process. As mentioned earlier, the mixing of nanoparticles in the matrix polymer is an important and challenging aspect due to the high aspect ratio of nanoparticles. The particles should be uniformly dispersed with a minimum amount of agglomeration to benefit from the composites. The initial tests will be conducted using manually mixed powder specimens due to the lack of immediate availability of resources. One of the other mixing methods identified from the literature will be used for subsequent experimental work. An extrusion process was used by Koo et al. [17] to produce a polyamide/clay nano-composite. A gravity-fed twin screw extruder was used to achieve the proper dispersion of nanoparticles in the study. A similar melt mixing process was used by Goodridge et al. [15] to produce a polyamide 12/CNT composite. The melt mixing procedures are followed by a cryogenic blending process to produce the required polymer nano-composite powder. This is conducted by basking the extruded material in liquid Nitrogen for five to ten minutes, followed by blending in a food processor.

Neat Polyamide 11 (PA11) was used as the matrix polymer, as it is a proven material for selective laser sintering and due to availability. Al_2_O_3_ powder particles with an approximate diameter of 50 nm were used as the filler material. Powder specimens were produced with Alumina compositions of 1 wt %, 2 wt %, 3 wt %, 5 wt %, and 10 wt % to investigate the effect of Alumina composition on the sintering process. The powder specimens were manually mixed as solid particles.

The specimens were sintered using an experimental Selective laser sintering setup, which allows the sintering conditions and material systems to be varied with minimal restrictions and produce samples using a minimum amount of material. A CO_2_ laser is used with a wave length of 10.6 um, which is ideal for processing polymer components. Both polyamide and Alumina have high absorptivity values, 75% and 96% respectively, for a CO_2_ laser wave length [10].

## 3. Initial Powder Characterization and Laser Sintering Results

### 3.1. Thermal Characterisation of the Raw Materials

**Figure 1:**
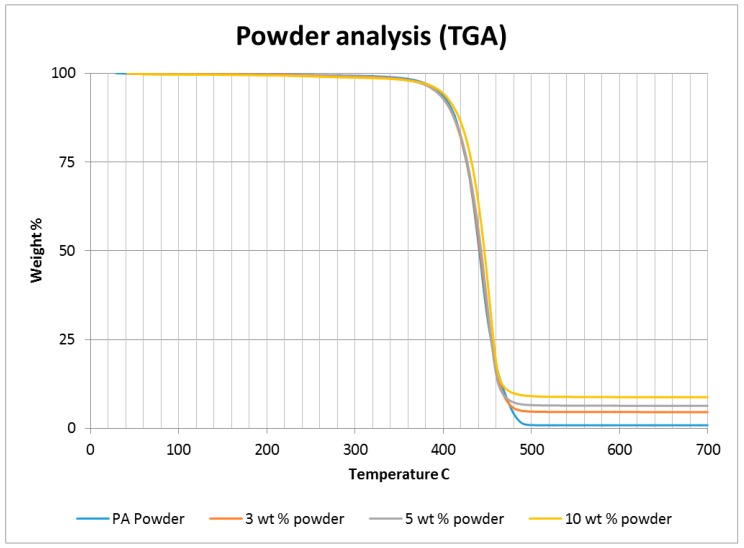
Thermo-gravimetric analysis (TGA) results based on the polymer nano-composite powder.

A thermo-gravimetric analysis (TGA) was conducted on the powder and sintered samples to identify the thermal degradation characteristics of the materials. The samples were heated from 30 °C to 700 °C at a rate of 10 °C/min in a TA instruments Discovery TGA system to achieve complete decomposition of PA11. The process was conducted in an air atmosphere and the percentage weight loss was plotted against the temperature and presented in Figure 1 using powders of varying compositions. The TGA results of the neat polymer presented in Figure 1 show an initial reduction in weight down to approximately 99.7% when heated from 90 °C to 110 °C, which can be identified as an evaporation of the moisture stored in the PA. The powder produces a graph characteristic of polyamide, as observed from the literature. A reduction in mass could be identified while heating in the range 400 to 480 °C. The decomposition temperature of the polymer can be identified by producing a derivative graph. This is identified as 443.524 °C at a decomposition rate of 2.55264 (wt %)/(°C). The graph indicates that the polymer powder is completely burned with a residual mass of 0.901276%.

An addition of 3 wt % alumina resulted in minor increases in both the decomposition (445.818 °C) and melting (186.442 °C) temperatures as depicted in Figure 1. The TGA curve obtained based on the 5 wt % alumina sample presented in Figure 1 shows the decomposition temperature to increase to 449.373 °C, and a rise in the residual content to 6.37% as a result of the increased alumina content. Figure 1 indicates a continuation of this trend, with an increased decomposition temperature of 453.222 °C and a residual mass of 8.79% when the alumina content was raised to 10 wt %.

**Figure 2:**
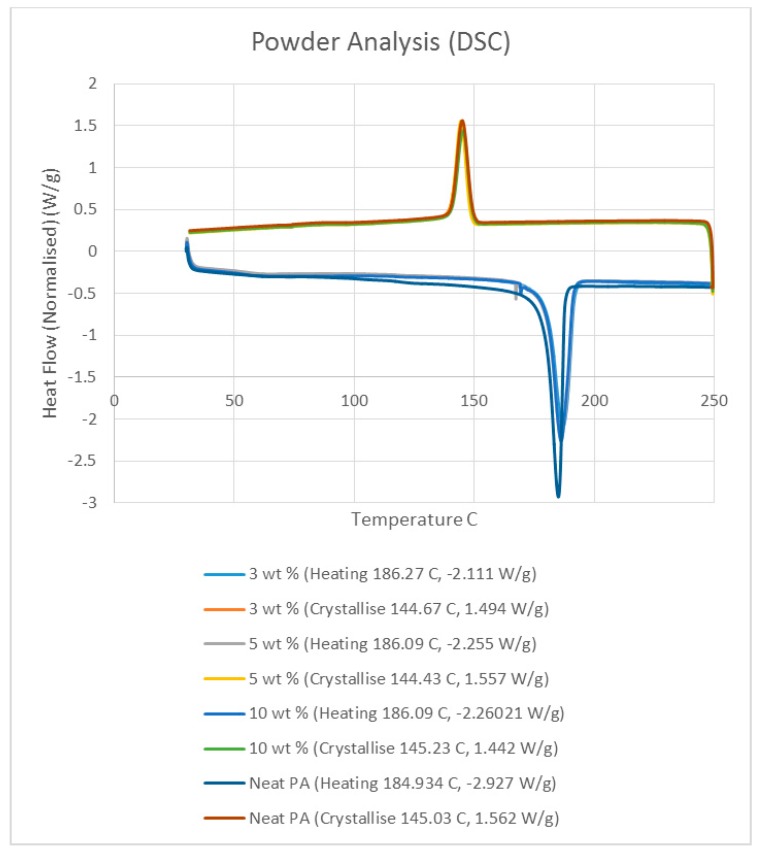
Differential scanning calorimetry (DSC) results based on the polymer nano-composite powder.

Differential scanning calorimetry (DSC) was done on all of the specimens using the temperature range identified prior to polymer decomposition. The samples were subjected to two heating cycles and a cooling cycle in the range of 30 °C to 250 °C at a rate of 10 °C/min in a nitrogen environment with a feed rate of 10 L/min. The results of the DSC tests are presented in Figure 2. As it may be observed from Figure 2, for the case of the neat polyamide, the melting temperature was noted to be 184.918 °C from the initial heating cycle of DSC, while the crystallization temperature was 145.025 °C. The secondary heating cycle yielded a lower melting temperature at 179.525 °C, indicating a deterioration of the polymer from the previous heating cycle. The addition of a nano alumina content of 3 wt % increased the melting temperature to 186.442 °C, as seen in Figure 2. The crystallization temperature was reduced to 144.675 °C, showing no major variation in thermal properties after the addition of the nano filler.

The characteristic curves showed a similar trend, indicating that the chemical structure of the matrix polymer remains unaltered during thermal processing when mixed with a given filler. The enthalpy for the melting process is reduced by the addition of the nano particles, indicating a lesser heat requirement to achieve melting. The trend continues with the melting temperature increasing to 186.095 °C, indicating an increase in heat absorption due to the higher alumina content at 5%. Overall, the temperature variations are minor with varying compositions, but a significant variation in the crystallization enthalpy may be noted between the neat polymer and the composites. The enthalpy of crystallization first increased by almost 50% when 3% nanoparticles are added to the neat polymer. However, with further increase in the nano alumina to 5% and 10%, the enthalpy of crystallization decreased below that of the neat polyamide. The enthalpy for the crystallization process is also known as the latent heat of crystallization, which is the amount of energy released to transform from a liquid state to a solid state.

While cooling neat polyamide of a given mass, a certain energy is released at the point of crystallization, corresponding to the peak shown on the cooling curve of Figure 2. With increased amounts of Al_2_O_3_ content, the relative amounts of latent heat should have been lesser. However, the nano Al_2_O_3_ particles, being more laser absorptive, gather relatively higher heat energy while heating. This heat is also not immediately conducted due to the presence of polyamide particles. Some of this heat is released during the cooling cycles of the polyamide-Al_2_O_3_ composite powders, compensating for some of the reduction in the energy released from the polyamide content. This is probably the reason why the peaks at crystallization are almost at the same level for samples of varying compositions.

### 3.2. Initial Laser Sintering Trials

The results of the DSC and TGA tests were used to evaluate the potential for laser sintering where the polymer and polymer composites are thermally stable for the laser sintering process. The clear melting and crystallization peaks, and the narrow but not overlapping range between the peaks indicated by the DSC graphs, suggests that the polymer and composite samples are suitable for laser sintering. The energy requirement for producing preliminary samples was estimated using temperature values obtained by DSC. The scan lines were simplified as cylindrical paths, while the density of the nanoparticles was neglected for a simplified initial estimate. The calculations were conducted for a laser beam diameter of 0.54 mm, polymer density of 1.04 × 10^3^ kg/m^3^, and a specific heat value of 1700 JK^−1^ kg^−1^. A bench mark scan speed of 500 mm/s was used for the calculations, which is a medium speed governed by the SLS system. Using the DSC results together with the basic heat transfer principles, the energy requirement for sufficient sintering was evaluated to be 9.5 W.

Single-layer samples were produced according to the American Society for testing Materials (ASTM) standards using predicted energy densities, while varying the laser power and scan speeds. The samples were subjected to thin film tensile testing to evaluate the mechanical properties of the composite material. Further testing was conducted using a range of energy densities to achieve better tensile properties [13]. The dispersion of the nanoparticles was analyzed by SEM and EBSD methods. The crystallinity and the rate of crystallinity of the composite can be evaluated by differential scanning calorimetry as shown by Kim et al. [18]. The thermal stability of the composite can be evaluated using thermo-gravimetric testing [19]. The thermal stability is a deciding factor for the repeatability of the process. Small rectangular specimens were produced using a range of laser power outputs and scan speeds within the successful energy density levels to determine preliminary sintering characteristics in the multi-layer samples [15]. The samples were then evaluated with SEM to determine the consolidation of material and predict the energy density requirements.

The polymer powder particles were in a non-melted state when sintered at 6 W with an Alumina content of 1 wt %. Signs of solid state diffusion could be identified due to neck formation among adjacent particles. The melt flow of polymer increased with increasing Alumina content under the same conditions. This can be seen by the developed scan lines in the sintering direction. A small amount of inter scan line coalescence can be seen at the 3 wt % loading. The interscan line melt flow decreased when the scan speed was increased while maintaining the same laser power. The melt flow of the polymer was significantly increased when the laser power was increased to 9 W. Clearly formed scan lines can be observed and the fusion between scan lines is improved at 9 W/1000 mm/s with a nanoparticle content of 3 wt %. The sintering behaviour is clearly improved with increasing nanoparticle content.

**Figure 3:**
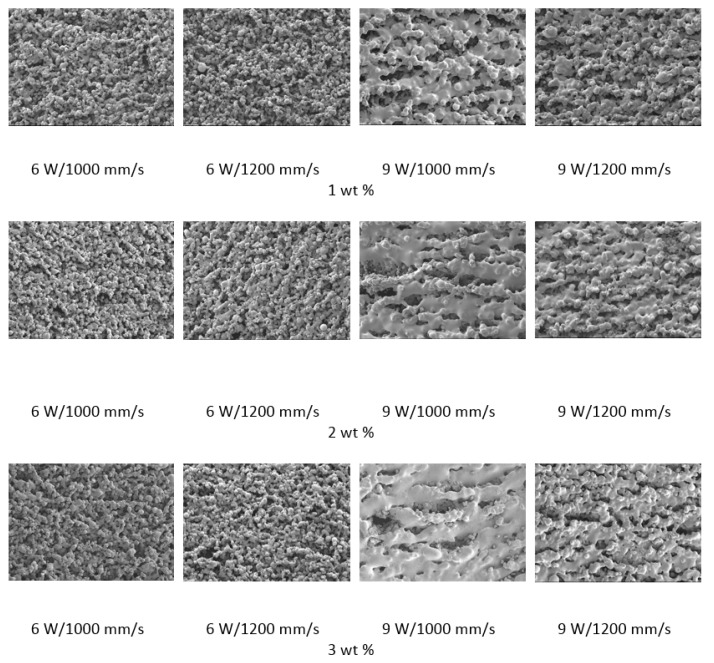
Photomicrographs of sintered Polyamide (PA)/nano Alumina preliminary trials.

**Figure 4:**
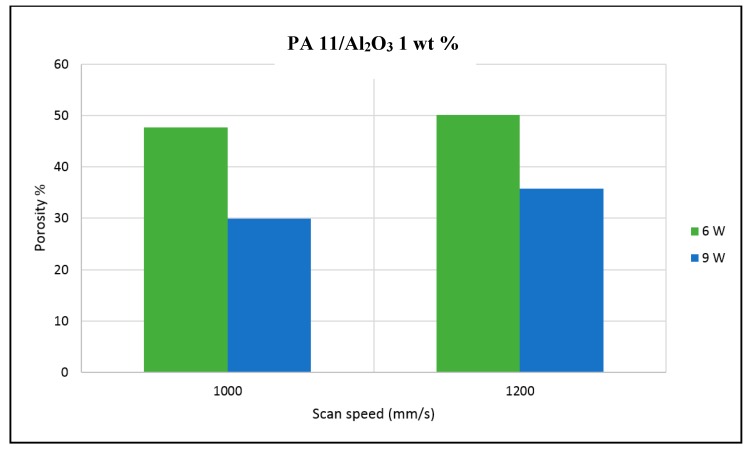
Polyamide 11 (PA11)/nano Alumina 1 wt % preliminary trial porosity analysis.

**Figure 5:**
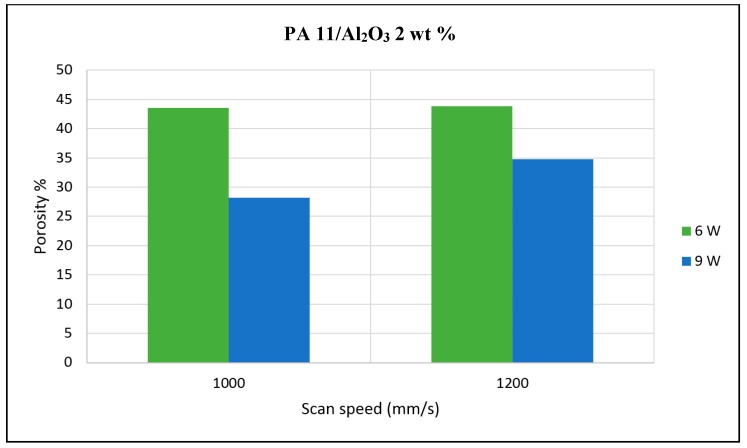
PA11/nano Alumina 2 wt % preliminary trial porosity analysis.

**Figure 6:**
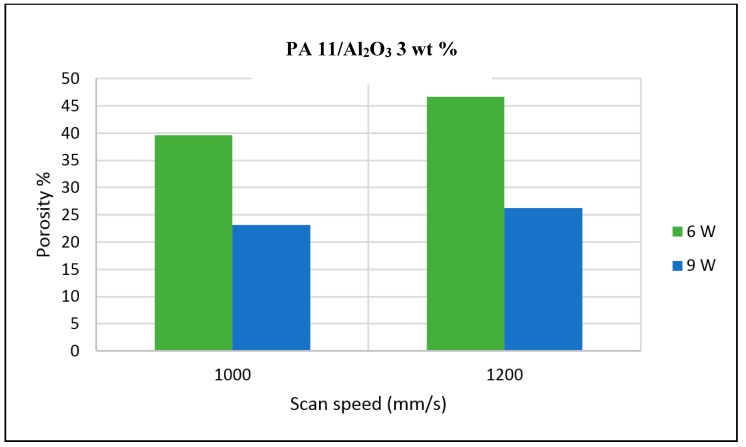
PA11/nano Alumina 3 wt % preliminary trial porosity analysis.

The porosity of the sintered parts were measured using an image analysis software, which identifies the voids in SEM images of single-layer samples. The porosity results support the observations made above, as the highest porosity is seen at the smallest loading condition as seen in Figure 3 and Figure 4, and the lowest porosity is achieved at the highest loading condition under high laser power and low scan speed. The porosity reduction at a higher loading condition is potentially due to the higher laser absorptivity of alumina compared to the matrix polymer, which increases the efficiency of the sintering process. The energy absorbed from the nanoparticles is also released to the surrounding polymer particles, as the operating temperatures are not sufficient to melt the alumina particles. This helps maintain the polymer particles at an elevated temperature for an extended duration, resulting in better sintering. The SEM morphology results and the porosity results from Figure 3, Figure 4, Figure 5 and Figure 6 suggest that material consolidation is higher at a laser power of 9 W and a scan speed of 1000 mm/s. The energy density for this configuration was calculated as 16,666 J/m^2^ according to Equation (1):

(1) $$\mathrm{Energy}\;\mathrm{Density}=\frac{\mathrm{Laser}\;\mathrm{Power}}{\mathrm{scan}\;\mathrm{spacing}\times \mathrm{scan}\;\mathrm{speed}}$$

The previous literature on Polyamide (PA) suggests that the elongation properties of sintered PA specimens significantly improve when the laser energy density is increased to 25,000 J/m^2^ [19]. Hence, the higher energy density was used for producing the Al_2_O_3_ samples.

## 4. Results Based on Sintered Specimens and Discussion

**Figure 7:**
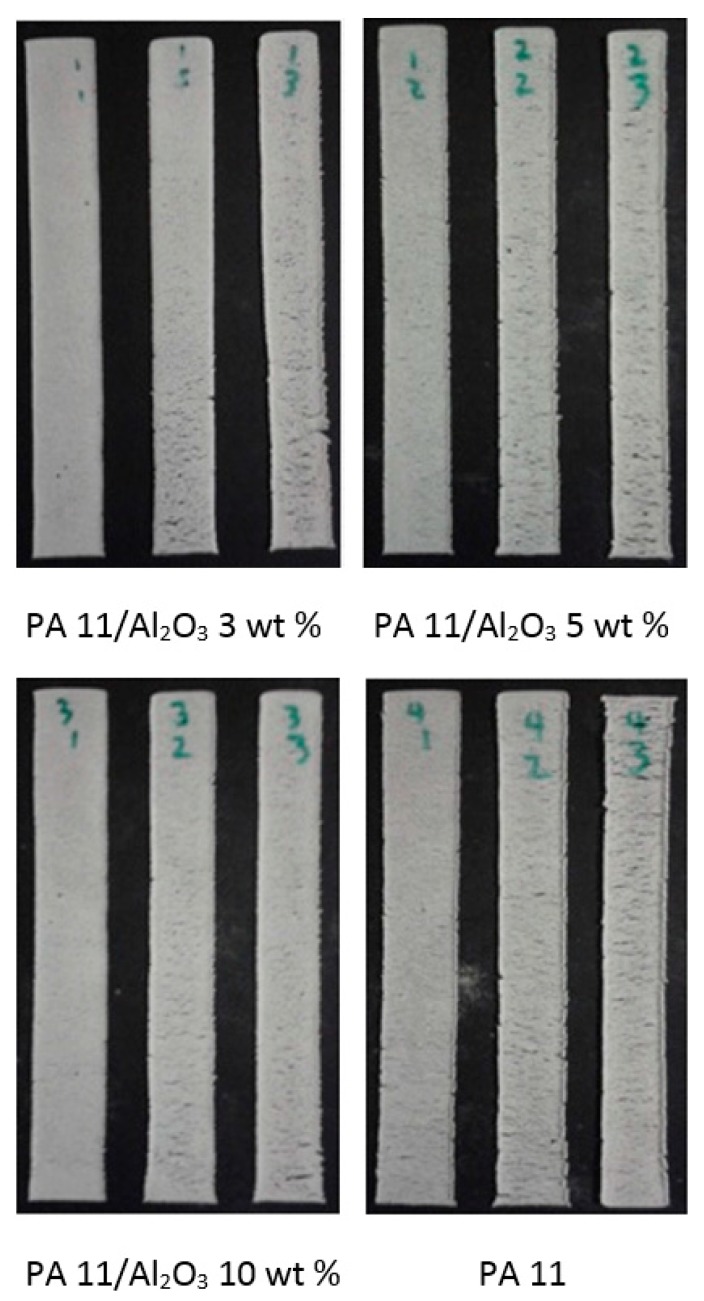
Sintered thin film tensile test specimens PA 11/Al_2_O_3_ 3 wt %, PA 11/Al_2_O_3_ 5 wt %, PA 11/Al_2_O_3_ 10 wt %, and Neat PA11 with laser power and speed giving rise to the same energy density 25,000 J/m^2^.

Thin film specimens were produced using the process ranges identified from the preliminary specimens as seen in Figure 7. The energy density was maintained constant at 25,000 J/m^2^, and laser power/speed configurations of 6 W/444 mm/s, 9 W/667 mm/s, and 12 W/889 mm/s were used. The samples were produced according to the ASTM D882 standard, and three repetitions of each sample were produced for each combination of parameters. The sintered specimens were subjected to SEM analysis to evaluate the mesostructure and the rheology corresponding to varying SLS parameters and material configurations. FTIR analysis was conducted to evaluate changes in the chemical composition of the sintered samples. TGA and DSC analyses were conducted to evaluate the thermal stability. The ASTM samples were subjected to thin film tensile parameters.

### 4.1. Morphologies of the Single-Layer Samples

**Figure 8:**
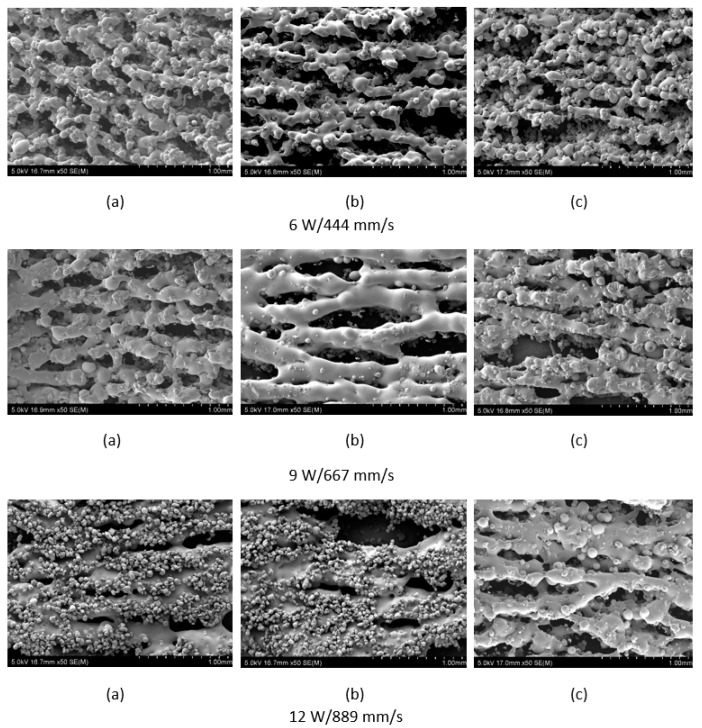
SEM analysis of (**a**) Polyamide + 3% nano Al_2_O_3_, (**b**) Polyamide + 5% nano Al_2_O_3_, and (**c**) Polyamide + 10% nano Al_2_O_3_ sintered thin film specimens processed at 6 W/444 mm/s (**top**), 9 W/667 mm/s (**mid**), and 12 W/889 mm/s (**bottom**); (**a**) 3 wt %; (**b**) 5 wt %; (**c**) 10 wt %, Al_2_O_3_, 50×, 5 kV.

The specimens showed an improvement in melt flow and material consolidation with increasing laser power despite the increase in scan speed when the energy density is maintained constant, as seen in Figure 8, indicating that the laser power has a higher significance on the variance of sintering properties. The SEM images also indicate that free polymer particles were attracted by the melt pool during the sintering process, which are partially consolidated. This phenomena is more visible at higher power. At 6 W, the polymer particles were in a partially melted state with some evidence of neck growth along the scan lines, but showed a lack of melt flow between the scan lines. This improved when the power was increased, and showed evidence of achieving near complete consolidation at 12 W. The SEM analysis conducted on sintered specimens with a filler content of 5 wt % showed a similar trend in the variation of consolidation with increasing laser power and speed, as seen in Figure 8b, but the sintering of polymer was less successful compared to the 3 wt % specimens. The attraction of solid particles to the melt pool is also visible at the tested polymer content. Figure 8c indicates that the consolidation of polymer was improved at an alumina content of 10 wt %, producing a similar trend to the previous loading conditions. The attraction of unsintered particles to the melt pool was less evident with an Al_2_O_3_ content of 10 wt %, in contrast to the previous loading conditions, indicating better thermal stability during the process.

### 4.2. FTIR Analysis on Sintered Specimens

**Figure 9:**
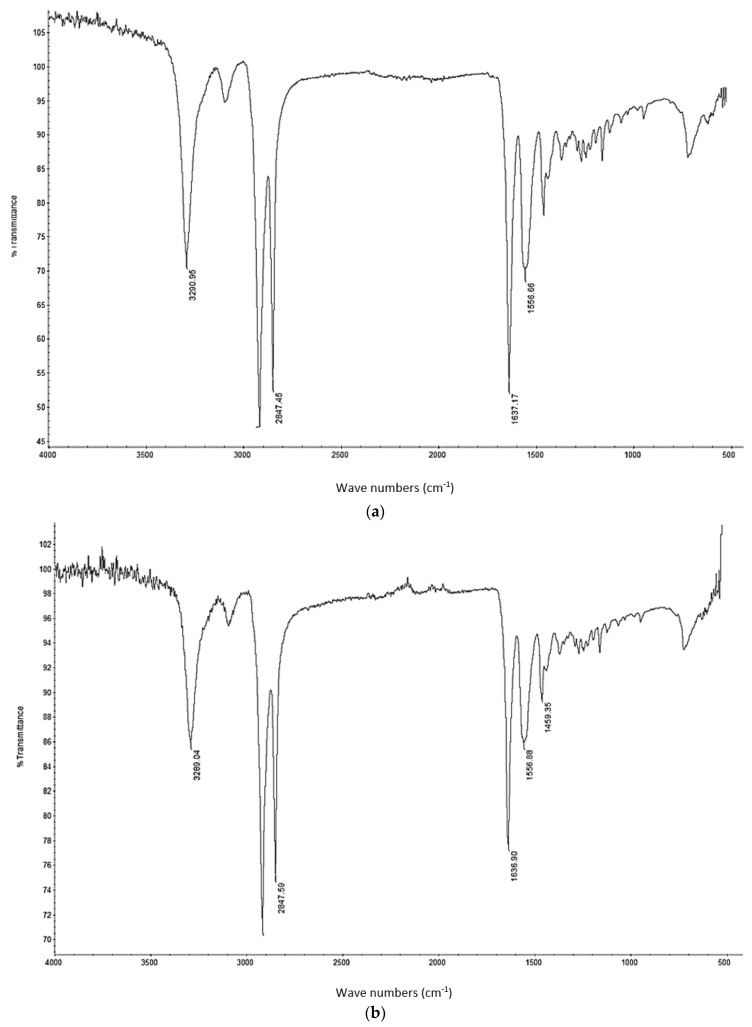

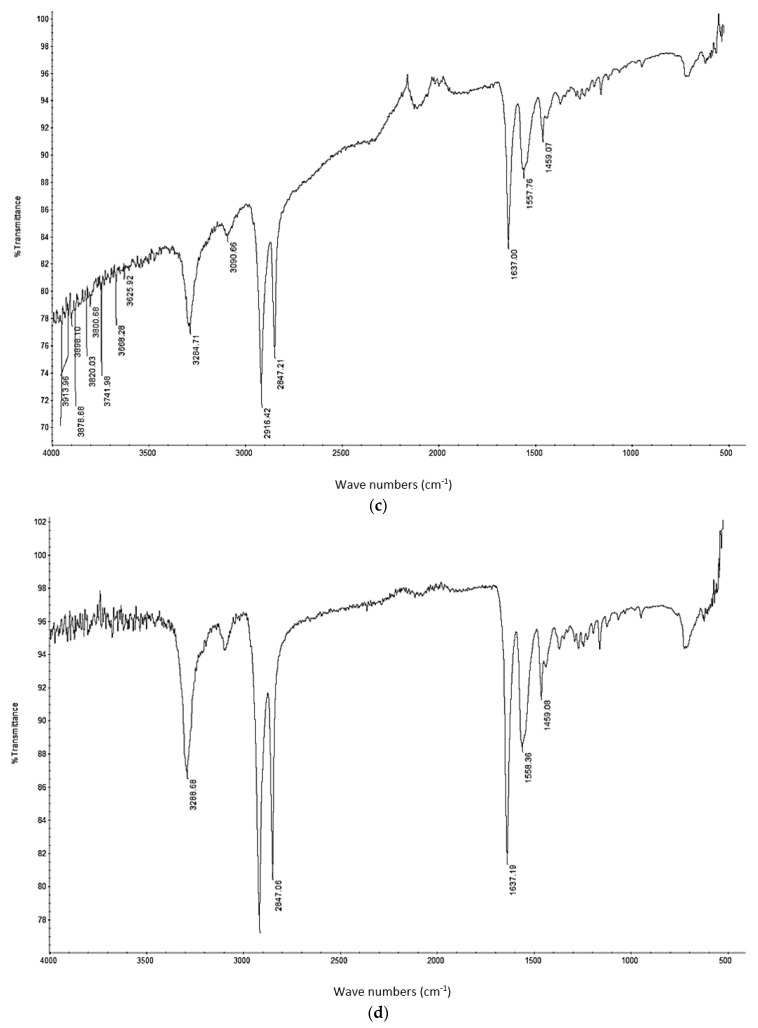
Fourier transformation infrared analysis of (**a**) Sintered neat polyamide; (**b**) PA11/Alumina 3 wt %; (**c**) PA11/Alumina 5 wt %; and (**d**) PA11/Alumina 10 wt %.

A Fourier transformation infrared analysis conducted on the sintered specimens was used to evaluate chemical changes due to heating and interactions with nano Al_2_O_3_ particles. From Figure 9, it can be seen that the chemical structure has remained intact, as the graphs indicate wave lengths corresponding to the neat polymer specimen. The wave lengths in the region of 3290 cm^−1^ correspond to the C–H bonds, while the peaks in the range of 2847 cm^−1^ correspond to H-C=O bonds in Polyamide. The other prevalent peak in the range of 1637 cm^−1^ indicates the N–H bonds. The results indicate that these chemical structures have remained intact after processing with nano Al_2_O_3_ particles. The minor peaks seen in Figure 9c are a result of noise produced during the test.

### 4.3. Thermal Analysis of Sintered Neat PA Specimens

**Figure 10:**
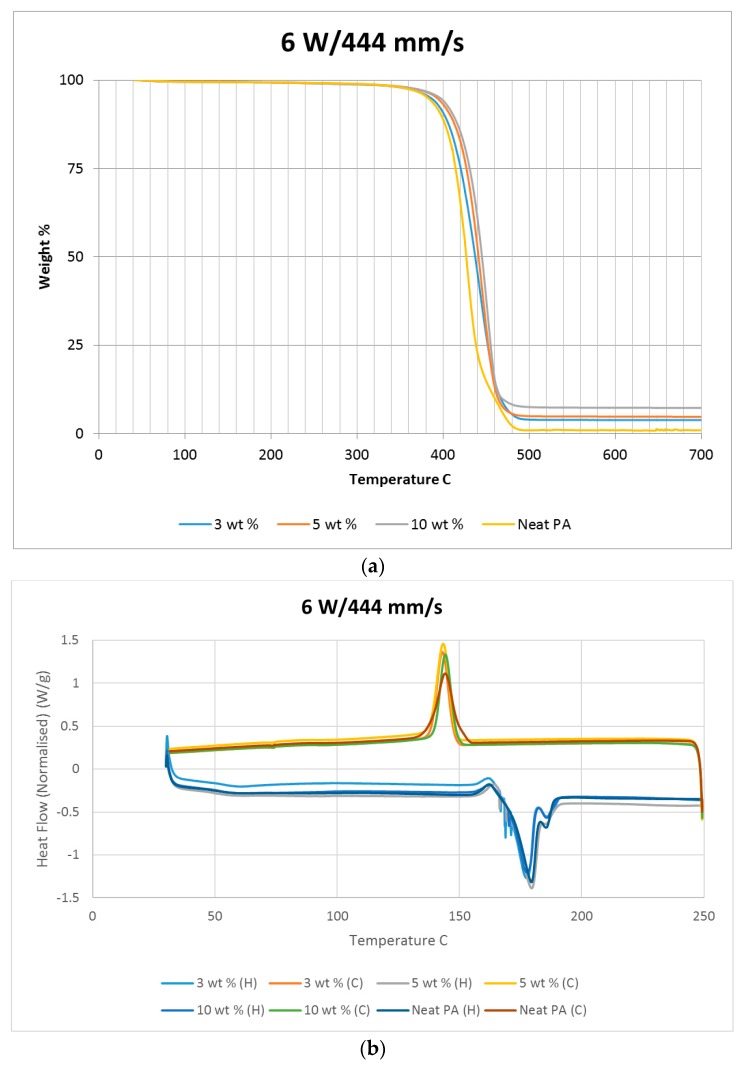
(**a**) TGA and (**b**) DSC results based on neat sintered samples at 6 W/444 mm/s.

The sintered PA neat samples showed a slight reduction in the rate of decomposition, as seen in Figure 10. This was not observed with other sintered samples mixed with nano alumina powder. This is potentially caused by a crystalline solidification of sintered neat polyamide. The neat polyamide powder also showed a slight variation similar to the sintered sample, as seen in Figure 1, which was not seen with the PA/Al_2_O_3_ composite powder. Considering the TGA results, it may be noted that for a given composition, varying power and speed settings should give rise to similar results when the same energy densities are used. This may be observed from the decomposition temperature results. However, the average decomposition temperature gradually increased with the amount of addition of Al_2_O_3_, which indicates a gradual increase in the stability of the polymer composite with an increasing wt % of Al_2_O_3_.

**Figure 11:**
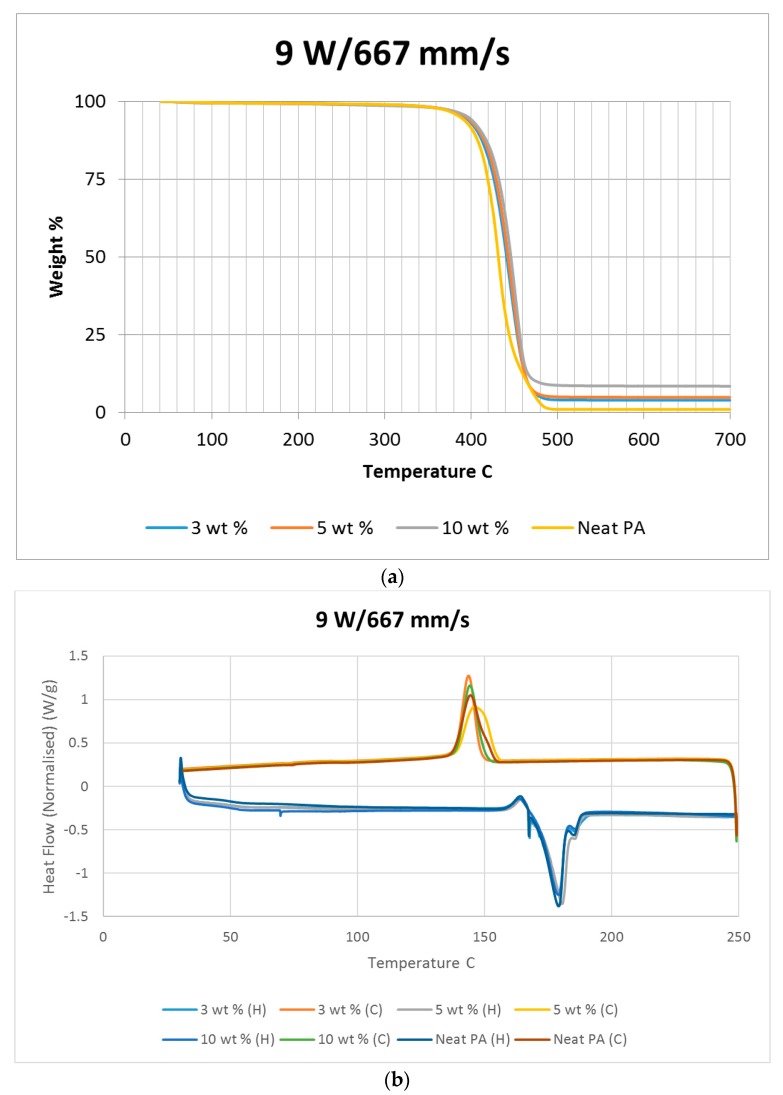
(**a**) TGA and (**b**) DSC results based on neat sintered samples at 9 W/667 mm/s.

**Figure 12:**
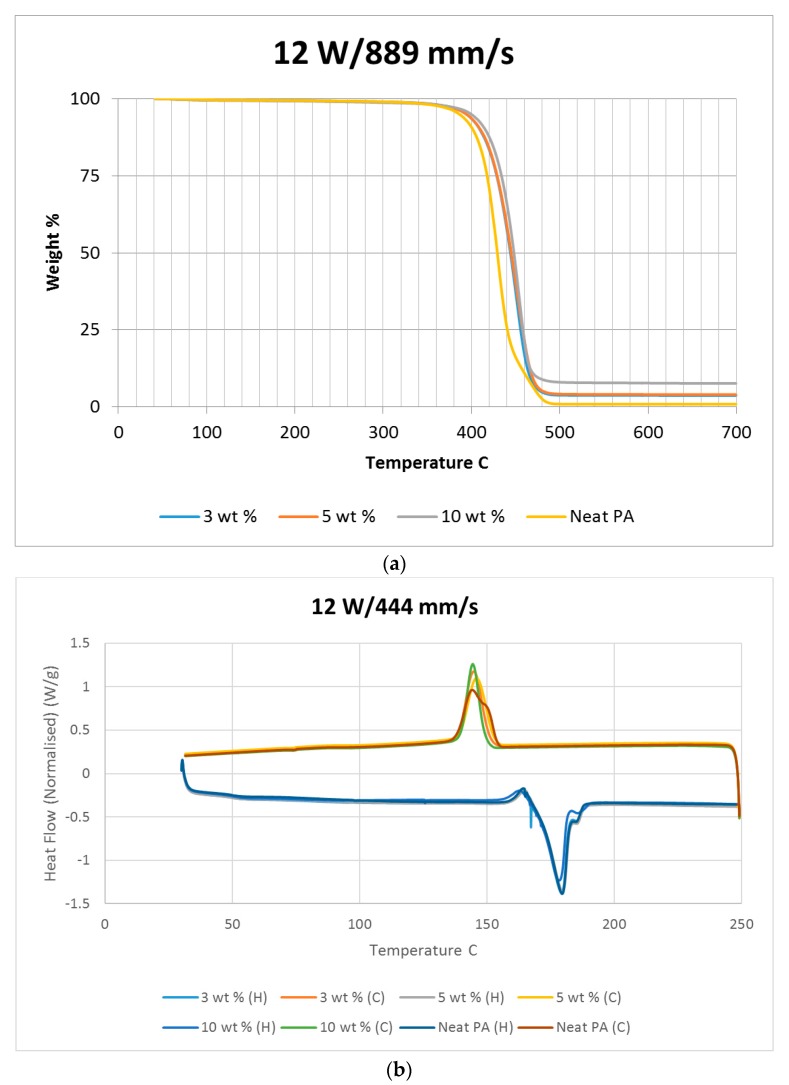
(**a**) TGA and (**b**) DSC results based on neat sintered samples at 12 W/889 mm/s.

The heat released while cooling the sintered polymer samples gradually reduced with increasing power settings, as noted in Figure 10. This could be indicative of a reduction in the rate of crystallization of the polymer due to exposure to higher temperatures. The PA11/Al_2_O_3_ 5 wt % composite shown in Figure 11b also showed a flatter cooling curve, representing a higher reduction in crystallinity compared to the other material combinations. Figure 10, Figure 11 and Figure 12 indicate a minimal variation in thermal characteristics between the composite configurations.

### 4.4. Tensile Testng

**Figure 13:**
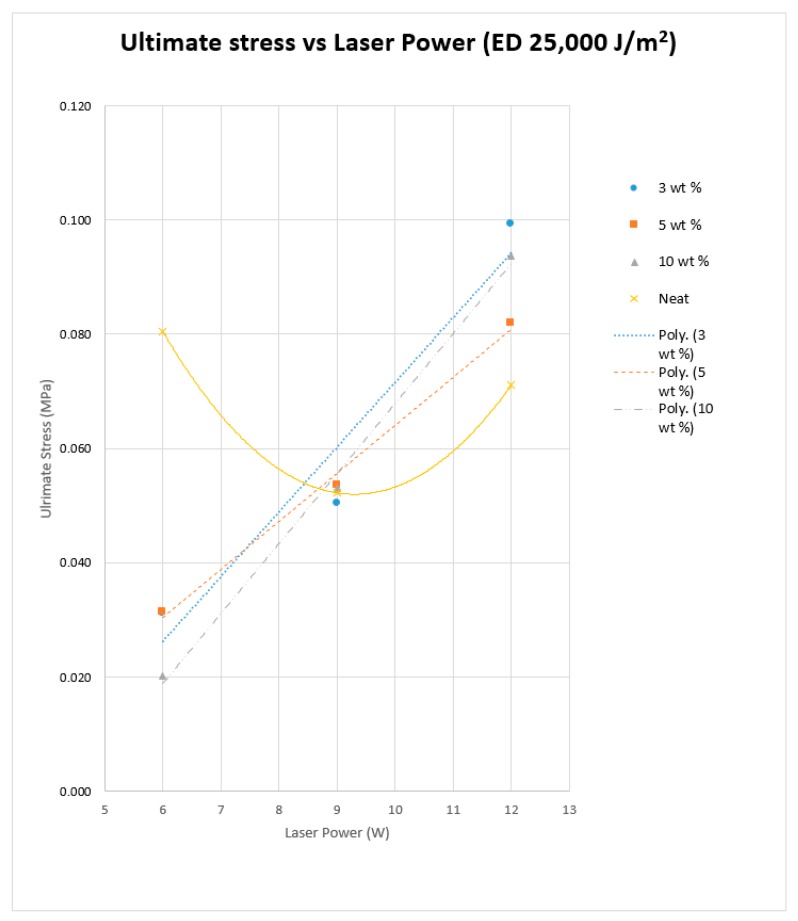
Average Tensile test results for sintered Neat Polyamide, and the Polyamide + 3% nano Al_2_O_3_, Polyamide + 5% nano Al_2_O_3_, and Polyamide + 10% nano Al_2_O_3_ thin film specimens.

**Figure 14:**
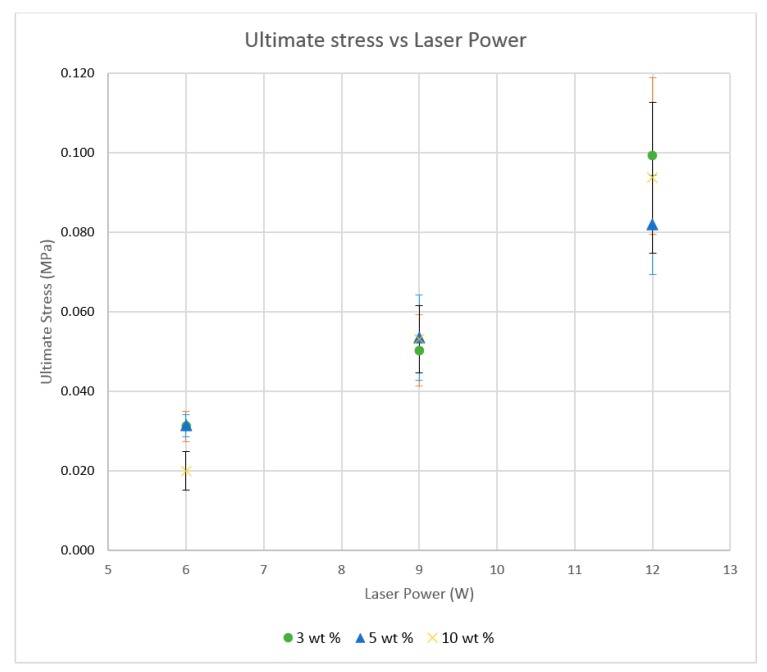
Tensile test result variations for three repetitions of the sintered Polyamide + 10% nano Al_2_O_3_ thin film specimens.

A fluctuating graph was identified as seen in Figure 13 for the trials conducted with neat PA11, which yielded the best tensile properties when sintered at low power and low speed (6 W/444 mm/s) while maintaining the same energy density. A dip in the PA11 tensile curve can be seen at 9 W, which corresponds to a configuration where the sintering time is inadequate at the given conditions. The nanoparticle-filled specimens showed a more linear variation of tensile strength with increasing laser power, which corresponds to an improvement in material consolidation, and indicates that the scan speed has a less significant effect on the process. The ultimate stress results provided a similar trend for all three composite materials, which is an increase in ultimate strength with increased laser power. The results show a reduction in reproducibility at higher laser power under reduced sintering times, as seen in Figure 14.

Figure 13 further indicates that the 3 wt % composite showed the most significant improvement in tensile strength with increasing laser power. The trend was closely followed by the 10 wt % composite. The ultimate stress for 5 wt % was less than that of the 3 wt % and 10 wt % composites. Al_2_O_3_ has a significantly higher laser absorptivity compared to PA powder, which allows the nanoparticles to absorb the laser energy more efficiently and release the heat into the polymer matrix for longer than the neat powder. The extended exposure to heat improves the solid state sintering of the polymer particles. This has an adverse effect at 6 W, as a significant amount of laser energy is absorbed by the ceramic powder, and the heat absorbed by the polymer is insufficient to reach the semi-solid rate required for proper coalescence. The powder coalescence and the tensile strength is improved with increasing laser power, and surpasses the Neat PA samples at 12 W/999 mm/s. This is due to the PA powder absorbing more energy both directly and indirectly (energy released into the matrix from ceramic particles) in a more efficient manner without being over exposed to high laser power, as seen in the case of neat PA at 12 W.

The presence of the ceramic nanoparticles within the matrix of the sintered base polymer may influence its mechanical characteristics, apart from altering the thermal fields of the substrate. The mechanical strength may be adversely affected due to the presence of the nanoparticles or groups of them together, leading to stress concentrations. This is evident in the mechanical strength decreasing as the amount of Al_2_O_3_ is increased from 3% to 5%. However, with further increase of the nano-ceramic component, the mechanical strength increased slightly, perhaps due to a favourable effect on the interparticle coalescence as evident from Figure 8. The excess energy absorbed is slowly released over time.

### 4.5. Elongation of the Samples

**Figure 15:**
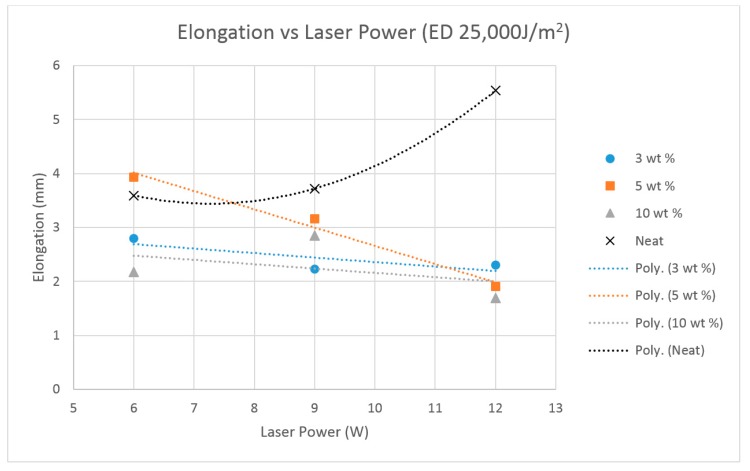
Average strain results for sintered Neat Polyamide, and the Polyamide + 3% nano Al_2_O_3_, Polyamide + 5% nano Al_2_O_3_, Polyamide + 10% nano Al_2_O_3_ thin film specimens.

The elongation results given by Figure 15 showed an opposite trend to the tensile strength in the alumina filled polymer, while the ultimate strain in sintered neat polyamide exponentially increased with increasing laser power despite maintaining a constant energy density. The reduction in elasticity is potentially caused by the nanoparticle agglomerates, which act as voids when subjected to a tensile load. The 3 wt % and 10 wt % composites showed a small gradual reduction in elasticity with reduced sintering time, while the 5 wt % composite indicated a significant reduction under the same sintering conditions.

## 5. Conclusions

Overall, the nano Al_2_O_3_ and polyamide composites are proved to be suitable for processing by laser sintering. The characterization results, however, showed varied responses with varying composition and process parameters. The following are the main conclusions based on the quantitative results:

- Based on the TGA and DSC results, the working laser power range of the PA/Al_2_O_3_ composite is established to be 6 to 9 W.
- The laser scan speed range is 1000 mm/s to 1200 mm/s.
- The optimum energy density for sintering the Al_2_O_3_/Polyamide powder composite is 25,000 J/m^2^.
- Material consolidation was improved when Al_2_O_3_ content was increased from 3 wt % to 10 wt %, and the best consolidation is achieved at a laser power output of 12 W.
- The DSC results indicated a small reduction in the rate of crystallization of sintered composite material compared to the neat powder and neat sintered specimens.
- The tensile strength of the sintered samples increased from the range of 0.2–1 MPa as the laser power is increased from 6 W to 12 W at the same energy density.
- The elongation of the sintered samples decreased when the laser power was increased, with the best results achieved with an Al_2_O_3_ content of 5 wt % at 6 W, giving an ultimate strain value of 0.28 mm/mm as against the lowest value of 0.07 mm/mm attained at 12 W.
